# Removal of DDT, DDD, and DDE from washing extracts of contaminated soil using polyaniline/bagasse composite material

**DOI:** 10.55730/1300-0527.3731

**Published:** 2025-04-12

**Authors:** Hop Quang NGUYEN, Bach Xuan NGUYEN, Thien Quang TRAN, Anh Van NGUYEN

**Affiliations:** 1 Department of Organic Chemistry, Faculty of Chemistry, Hanoi Pedagogical University 2 (HPU2), Vinh Phuc, Vietnam; 2 Department of Physicochemistry, Faculty of Chemistry, Hanoi Pedagogical University 2 (HPU2), Vinh Phuc, Vietnam; 3 Department of Food Safety and Quality Management, Faculty of Food Science and Technology, Ho Chi Minh City University of Industry and Trade, Ho Chi Minh City, Vietnam

**Keywords:** Polyaniline, bagasse, adsorption, plant protection drugs, DDT

## Abstract

Polyaniline (PANi) was hybridized with a bagasse (BG) substrate to treat Dichlorodiphenyltrichloroethane (DDT), dichlorodiphenyldichloroethylene (DDE), and dichlorodiphenyldichloroethane compounds, which are persistent organic pollutants (POPs) causing environmental contamination. The PANi/bagasse (PA/BG) composite was synthesized using ammonium persulfate and sulfuric acid, achieving efficiencies ranging from 82.63% to 86.92% with different ratios of PANi monomer to BG. Infrared spectroscopy (IR) and scanning electron microscopy (SEM) were used to characterize the synthesized materials. The adsorption capacities of DDT, DDD, and DDE compounds were investigated under various conditions, including adsorbent type, adsorption time, adsorbent dosage, and adsorbate concentration. Both Langmuir and Freundlich isotherm models were applied to evaluate the adsorption process, and the results indicated that both models were suitable for describing the adsorption of DDT, DDD, and DDE by the PANi/bagasse composite material.

## 1. Introduction

The plant protection drugs previously used in Vietnam and around the world were largely persistent organic pollutants (POPs), leading to environmental contamination and significant impacts on human health today. Dichlorodiphenyltrichloroethane (DDT), dichlorodiphenyldichloroethylene (DDE), and dichlorodiphenyldichloroethane (DDD) are among these POPs, which have contaminated the environment and have severely affected human health, causing health problems such as cancers, neurological disorders, reproductive issues [1–3].

In Vietnam, environmental pollution caused by pesticides remains a severe issue. According to statistics from the Ministry of Natural Resources and Environment of Vietnam, there are currently over 1556 locations (villages and communes) where agricultural land is contaminated with pesticides, particularly in the central provinces such as Thanh Hoa, Nghe An, Ha Tinh. As a result, an urgent requirement now is to treat and restore the contaminated land areas [4]. The National Environmental Status Report for the period 2016–2020 by the Ministry of Natural Resources and Environment indicated that while many sites with residual pesticide pollution had been treated, a significant number of contaminated sites have yet to be thoroughly addressed^1^

Currently, many methods have been applied to treat and reduce the pollution caused by persistent organic pollutants (POPs), such as biodegradation [5], advanced oxidation processes [6], electrochemical oxidation [7,8], Daramend technology 2, soil washing [9–11]. Among these, soil washing is supposed to be one of the most effective methods, which applies to all levels of contamination, even at high contamination levels, and in fact, it has shown great potential [9–14].

Following soil washing [10–12], the washing solutions must undergo treatment to remove contaminants, and the adsorption method is applied to recover and treat these pollutants. Adsorption is an effective method, using materials such as activated carbon, clay minerals, alumina, natural zeolites, biochar, and polymers to recover and treat pollutants [15–21].

Polyaniline is known as a low-cost material that is easy to synthesize, possesses excellent environmental stability, and also offers diverse and highly effective applications [16–21]. Furthermore, PANi has been used for the adsorption and treatment of environmental contaminants, including organic compounds and heavy metals [16–19,22–27].

In particular, PANi can be blended and combined with biomass sources, which are low-cost and readily available materials. This combination offers a high potential for the adsorption and treatment of environmental pollutants. Specifically, PANi has been combined with various types of biomass materials for the adsorption of heavy metals [23–27] and persistent organic compounds such as DDT, and polychlorinated biphenyls (PCBs) [16,27–29].

Bagasse is a very widely available and easily accessible biomass material in Vietnam, and it has been utilized for the removal of contaminants [30–32]. The combination of PANi with bagasse during the synthesis of PANi polymer results in the formation of PA/BG material, which is used for the adsorption and treatment of persistent organic pollutants extracted from the contaminated soil. Specifically, in this study, the PANi/bagasse (PA/BG) material which was evaluated for its effectiveness in adsorbing DDT, DDD, and DDE compounds, demonstrated high efficiency.

## 2. Materials and methods

### 2.1. Chemicals

Ammonium peroxodisulfate (Merck, 98%); sulfuric acid (Merck, 98%); aniline (Merck, 99.5%); acetone (Chemsol-VN, 99.7%); double-distilled water was distilled in the laboratory using an Aquatron A4000D apparatus from STUART, UK; the research solution contained DDT, DDD, and DDE compounds washed from contaminated soil and bagasse powder.

### 2.2. Analytical equipment

This study utilized Fourier Transform Infrared Spectroscopy (FT-IR) and Scanning Electron Microscopy (SEM) to elucidate the structural and morphological characteristics of polyaniline (PANi), bagasse (BG), and PANi/bagasse (PA/BG) composite materials. Furthermore, Gas Chromatography-Mass Spectrometry (GC-MS) was employed to analyze solutions containing DDT, DDD, and DDE compounds throughout the adsorption process.

FT-IR analysis was conducted using the KBr pellet technique for the material samples and a Thermo Nicolet Nexus 670 Fourier Transform Infrared Spectrometer (USA), covering the frequency range from 4000 to 400 cm^−1^, performed for 32 scans with a resolution of 4 cm^−1^; spectra were processed using Omnic 5A and Origin 9.0 software.

The surface morphology of the materials under vacuum conditions was examined using a Scanning Electron Microscope JOEL JSM-6510LV (Japan) with magnifications ranging from 5x to 300,000x (printed as a 128 mm × 96 mm micrograph).

Gas chromatography-mass spectrometry system GCMS – QP 2010 Plus from Shimadzu, Japan; Separation column DB-5ms EQUTI-5 with dimensions of 0.25 mm × 0.25 μm × 30 m; Centrifuge Rotofix 32A (Hettich Zentrifugen) from Hettich, with a maximum rotation speed of 4000 rpm; Ultrasonic cleaner S100 Elmasonic from ELMA, Germany, operating at an ultrasonic frequency of 37,000 Hz, with an ultrasonic power of 600 W and heating capability; Sample homogenizer mixer Vortex from the USA, capable of shaking speeds of up to 3000 rpm, with two operating modes: continuous and touch START/STOP; Vacuum rotary evaporator Stuart from the UK, with a rotation speed range of 20–190 rpm and a vacuum level of less than 1 mmHg; Solid-phase extraction system Bond Elut-C18 columns, containing 500 mg of sorbent with a volume of 3 mL, and the sorbent was silica with a particle size of 120 μm and pore size of 20 μm, from Agilent. The Bond Elut-C18 solid-phase extraction column system contained 500 mg of adsorbent, with a capacity of 3 mL, and the adsorbent was silica with a particle size of 120 μm and a pore size of 20 μm, manufactured by Agilent.

Additionally, the EPA CLP Organochlorine Pesticide Mix, Sigma-Aldrich (certified reference material, 2000 μg/mL each component in hexane/toluene = 1/1, 1 mL ampule), containing the compounds DDT, DDD, and DDE, was used as a standard for constructing the calibration curve in GC-MS analysis.

### 2.3. Synthesis of adsorbent materials

Bagasse was first collected from local sugarcane juice vendors, cut into small pieces, washed with distilled water at 70 °C, then dried at from 60 to 70 °C, and finally ground into a fine powder.

**Figure 1:**
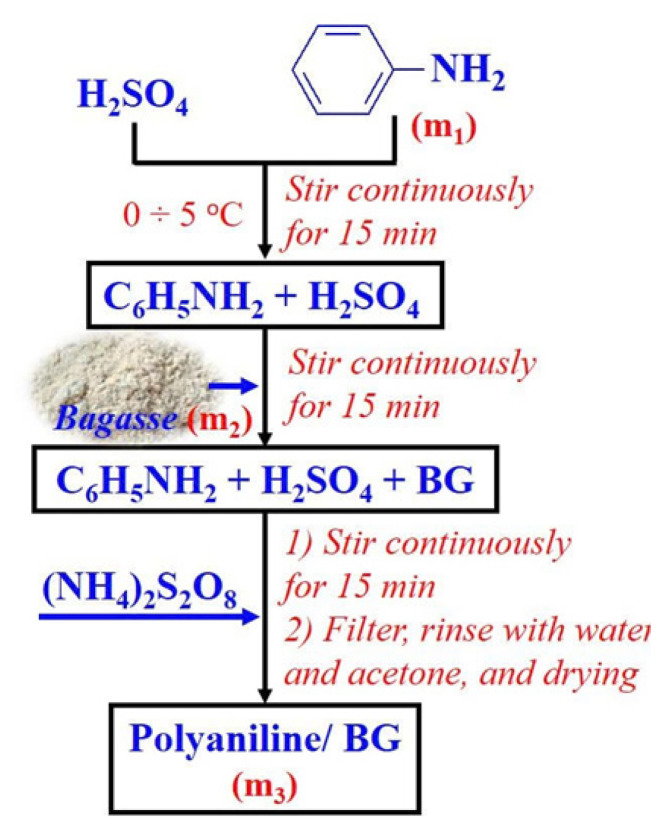
Synthesis of polyaniline/bagasse composite materials.

Four PANi/bagasse (PA/BG) materials were synthesized with varying initial mass ratios of aniline/bagasse (ANi/BG) at 1/0, 1/2, 2/1, and 1/1 (Figure 1). Specifically, the ratios were as follows: 1/0 (PANi without bagasse, denoted as PA/BG10), 1/2 (denoted as PA/BG12), 2/1 (denoted as PA/BG21), and 1/1 (denoted as PA/BG11). The PA/BG materials were synthesized through a chemical method, using (NH_4_)_2_S_2_O_8_ as the oxidizing agent in an acidic medium of H_2_SO_4_. The entire process was stirred using a magnetic stirrer for 15 h at a temperature range of 0 to 5 °C (cooled with ice water). After the synthesis, the PA/BG materials were washed once with dilute acetone and several times with distilled water until a neutral pH was achieved. Finally, the products were dried in an oven at 70 to 80 °C. The PA/BG materials were stored in airtight plastic containers and desiccators [28–29].

The synthesis yield of polyaniline/bagasse was calculated using the equation (1):


(1) $$\%\text{Yield}=\frac{{\text{m}}_{3}-{\text{m}}_{2}}{{\text{m}}_{1}}\times 100$$

Where: m_1_ is the initial mass of ANi, m_2_ is the mass of bagasse added during the synthesis process, and m_3_ is the mass of the synthesized PA/BG material.

### 2.4. Sample solution

**Figure 2:**
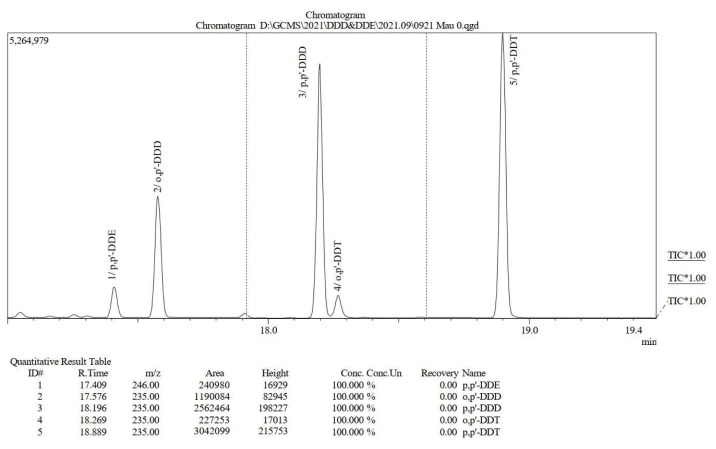
GC-MS chromatogram of the initial adsorption solution.

The solution used in this study contained the compounds *o,p′*-DDT, *p,p′*-DDT, *o,p′*-DDD, *p,p′*-DDD, and *p,p′*-DDE, which originated from the washing process of contaminated soil collected from the Hon Tro area, Dien Chau district, Nghe An province, Vietnam [10–12]. Before adsorption studies, the solution was analyzed for its compound contents using the GCMS analysis method (Figure 2) and the specific results are presented in Table 1.

Five different material samples were used: PA/BG10 (PANi without bagasse), PA/BG12, PA/BG21, PA/BG11, and bagasse (denoted as PA/BG01). Each sample weighed 0.5 grams to evaluate its adsorption capability for treating the washing solution obtained from the contaminated soil with the initial total DDT concentration of C_0_ = 1546 ppm and a volume of 20 mL. The experiment was conducted with continuous stirring using a magnetic stirrer for 10 h. After the experiment, 1.0 mL of each sample solution was transferred into a COD-16mm glass tube to analyze the remaining concentration in the solution after adsorption using the GC/MS method.

In addition, studies were conducted to evaluate the adsorption capacity of the PA/BG12 material for the compounds DDT, DDD, and DDE under various conditions, including changes in adsorption time, the mass of the adsorbent material, and the concentration of the adsorbates. All adsorption experiments were conducted on a magnetic stirrer at a speed of 100 rpm, under a constant room temperature of 25 °C. Upon completion of the experiments, a 1.0 mL aliquot of the solution was collected for concentration analysis using the Gas Chromatography-Mass Spectrometry (GC-MS) method.

### 2.5. Effect of adsorption time

The study investigated the effect of contact time on the adsorption process to determine the equilibrium time. Adsorption experiments were conducted at time intervals of 5, 10, 20, 40, and 80 min using 0.1 g of PA/BG adsorbent material. The sample solution had a total concentration of 120 ppm of DDT, DDD, and DDE compounds in a volume of 30 mL.

### 2.6. Effect of adsorbent mass

The influence of adsorbent mass on the adsorption process was assessed by varying the PA/BG adsorbent quantity (0.02, 0.04, 0.06, 0.08, and 0.1 g) to determine the optimal mass for maximum adsorption efficiency. These experiments were performed using a solution with a total concentration of 120 ppm, a volume of 20 mL, and an adsorption time of 120 min.

### 2.7. Effect of Initial DDT, DDD, and DDE concentrations

The effect of initial contaminant concentration on adsorption performance was evaluated at total initial concentrations of 50, 150, 200, 250, and 300 ppm. Adsorption studies were conducted using 0.1 g of PA/BG adsorbent and a solution volume of 20 mL. The adsorption capacity was analyzed, and the adsorption mechanism was characterized using the Langmuir and the Freundlich isotherm models. The results indicated that the substances present in the solution samples after adsorption were primarily the compounds o*,p′*-DDT, *p,p′*-DDT, *o,p′*-DDD, *p,p′*-DDD, and *p,p′*-DDE. The adsorptive capacity and adsorption efficiency of the material for these compounds were calculated using equations (2–3):


(2) $$\text{q}=\frac{({\text{C}}_{0}-\text{C})\text{V}}{\text{m}}$$

(3) $$\text{E}\%=\frac{\left({\text{C}}_{0}-\text{C}\right)}{{\text{C}}_{0}}\times 100$$

Where: q is the adsorption capacity (mg/g); V is the volume of the adsorbate solution (L); m is the mass of the adsorbent (g); E% is the adsorption efficiency; C_0_ and C are the initial and final concentrations of the adsorbate (mg/L).

### 2.8. Adsorption isotherm

The Langmuir and Freundlich isotherm models were used to describe the adsorption process of *o,p′*-DDT, *p,p′*-DDT, *o,p′*-DDD, *p,p′*-DDD, and *p,p′*-DDE compounds by the PANi/bagasse composite material at a constant room temperature of 25 °C.

The Langmuir adsorption isotherm model describes and explains that the molecules of the adsorbate are adsorbed in a monolayer and uniformly across the surface of the adsorbent. In contrast, the Freundlich model is an equation that describes adsorption occurring on a heterogeneous surface, not limited to a monolayer. The Langmuir and Freundlich adsorption isotherm equations were used to describe adsorption through the following equation (4–5)[33–37]:


(4) $$\frac{\text{C}}{\text{q}}=\frac{\text{C}}{{\text{q}}_{\text{max}}}+\frac{1}{{\text{q}}_{\text{max}}\times {\text{K}}_{\text{L}}}$$

(5) $$\text{Ln q}=\text{Ln }{\text{K}}_{\text{F}}+\frac{1}{\text{n}}\times \text{Ln C}$$

q_max_ is the maximum adsorption capacity per unit mass of adsorbent (mg/g), K_L_ (L/mg) is a constant characteristic for the Langmuir adsorption model; K_F_ (mg/g) and n are constants that characterize the Freundlich adsorption model.

## 3. Results and discussion

### 3.1. Synthesis and characterization of PANi/bagasse material

#### 3.1.1. Material synthesis efficiency

The synthesis yield of PANi materials with bagasse (Eq. 1), based on experimental data, specifically presented in Table 2, showed a high synthesis yield ranging from 82.63% to 86.92%.

#### 3.1.2. Fourier transform infrared spectrum (FT-IR)

**Figure 3:**
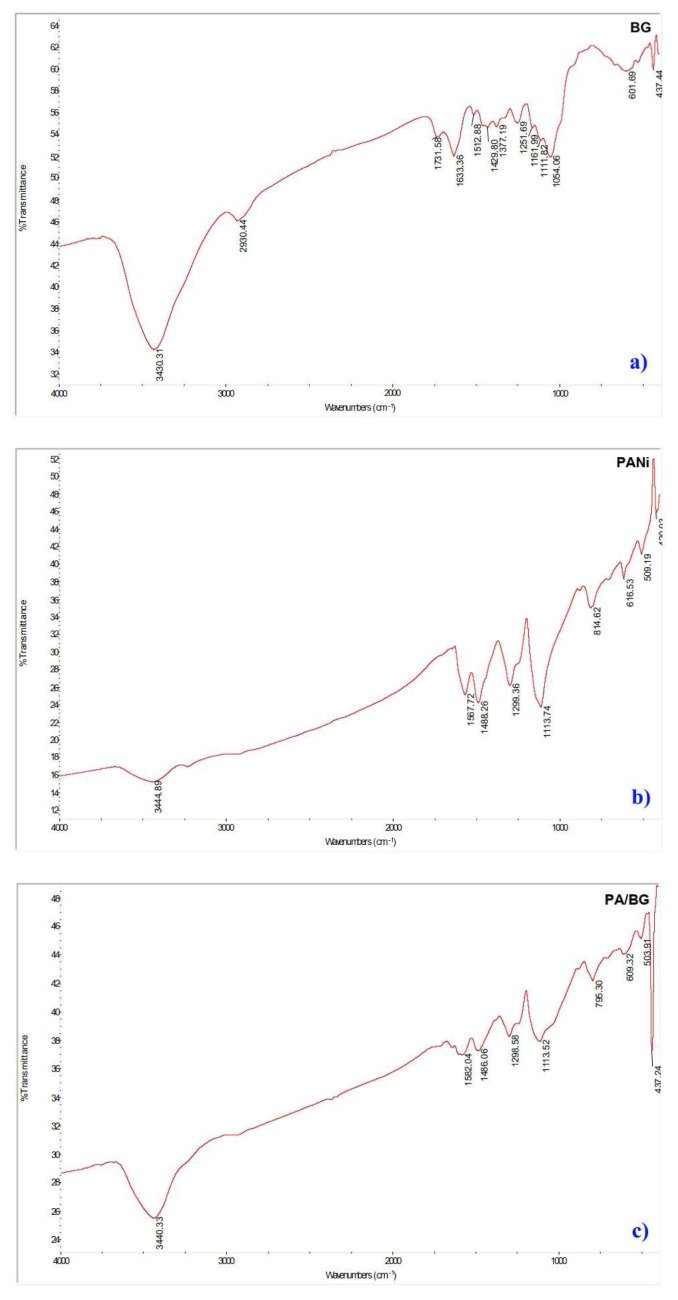
The infrared spectrum of bagasse (a), PANi (b), and PANi/bagasse (c).

Figure 3a shows the FT-IR spectrum of bagasse, indicating the characteristic vibrational signals corresponding to the cellulose, hemicellulose, lignin, and pectin present. Specifically, the spectrum exhibits the stretching vibrations of the -OH group at 3430.31 cm^−1^; the C–H stretching vibrations of methyl and methylene groups at 2930.44 cm^−1^; the stretching vibrations of the C=O bond in carboxylic groups at 1731.58 cm^−1^; the aliphatic C–H bending vibrations at 1377.19 cm^−1^; the stretching vibrations of the C–O bond in lignin at 1251.69 cm^−1^; the asymmetric stretching vibrations of the C–O–C bond in pyranose rings at 1161.99 cm^−1^, characteristic of cellulose and hemicellulose; and finally, the asymmetric C–O stretching vibrations of the glucosidic ring in cellulose and hemicellulose at 1054.06 cm^−1^.

The FT-IR spectrum data of PANi material in Figure 3b exhibits the characteristic features of PANi’s structure. The aromatic rings, such as the benzoid ring, show vibrational peaks at 1567.72 cm^−1^, and the quinoid ring in the diamine form shows vibrational peaks at 1488.26 cm^−1^. Additionally, the secondary amine group’s N–H stretching vibrations appear at 3444.89 cm^−1^. The nitrogen atoms double-bonded to the quinoid ring (N = quinoid = N) exhibit vibrations at 1299.36 cm^−1^. Furthermore, the stretching vibrations of the C–N^+^ and C-H groups are observed at 1113.74 cm^−1^ and 814.62 cm^−1^, respectively.

The FT-IR spectrum of PA/BG (Figure 3c) shows signals of the characteristic functional groups of PANi, along with some functional groups from sugarcane bagasse, which appear as weak signals and are not visible in the IR spectrum. Based on the spectrum results (Figure 3), it can be confirmed that the synthesis of the composite material between PANi and sugarcane bagasse has been successful. The signals of the characteristic functional groups for these materials are detailed in Table 3.

#### 3.1.3. Scanning electron microscope

**Figure 4:**
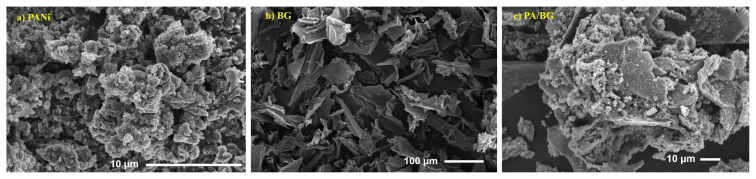
SEM image of PANi (a), bagasse (b) and PANi/bagasse (c).

The SEM images of PANi, BG, and PA/BG materials, captured using a scanning electron microscope (SEM) in Figure 4, show that the crushed bagasse has a fibrous structure, appearing as long threads, scales, or overlapping plates. PANi material appears as fibers, overlapping and porous, with nanofiber diameters ranging approximately from 50 to 80 nm. The SEM images of the PANi-based adsorbent material synthesized on bagasse (PANi/BG) resemble the morphology of bagasse, with PANi coating the bagasse, confirming that PANi has been successfully synthesized on the bagasse substrate.

### 3.2. Evaluation of adsorption capacity of PA/BG composite materials

#### 3.2.1. Effect of adsorbent materials

**Figure 5:**
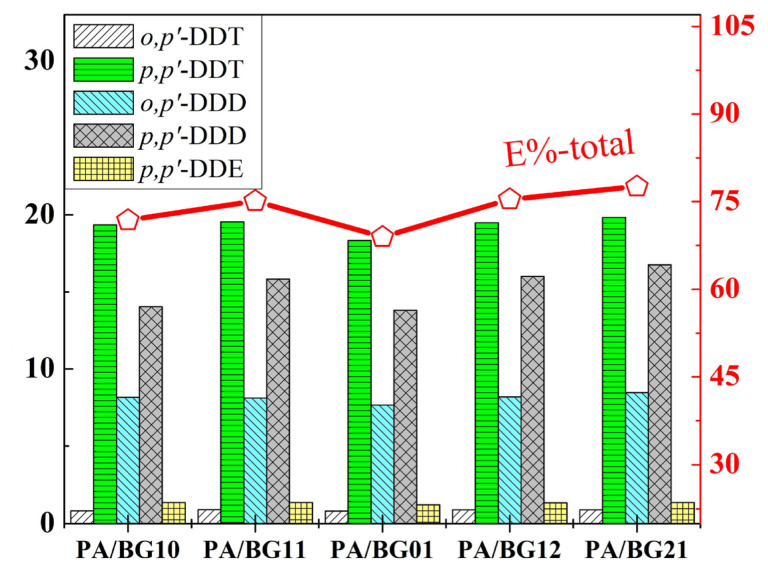
Adsorption capacity of different materials.

The adsorption capacities of the PA/BG composite materials with different mass ratios of ANi and bagasse are shown in Figure 5. The results indicate that all the materials used could adsorb DDT, DDD, and DDE compounds, achieving adsorption efficiencies ranging from 68.94 to 77.58%. Among these, the compounds *p,p′*-DDT and *o,p′*-DDD exhibited the highest adsorption capacities, while *o,p′*-DDT had the lowest adsorption capacity. This is consistent with the initial concentration ratios of the compounds in the solution.

Based on the results, it is evident that the composite materials PA/BG11 (q_total_ = 45.76 mg/g), PA/BG12 (q_total_ = 45.91 mg/g), and PA/BG21 (q_total_ = 47.28 mg/g) exhibit better adsorption capacities than the single materials PA/BG10 (polyaniline) and PA/BG01 (bagasse). This indicates that the combination of PANi and bagasse in the synthesis process results in superior adsorption performance compared to single materials. Therefore, to reduce the cost of synthesizing the adsorbent material, the material with a higher proportion of bagasse, PA/BG12, should be selected for further studies on the adsorption capacity of the composite material PA/BG under different conditions.

#### 3.2.2. Effect of adsorption time

**Figure 6:**
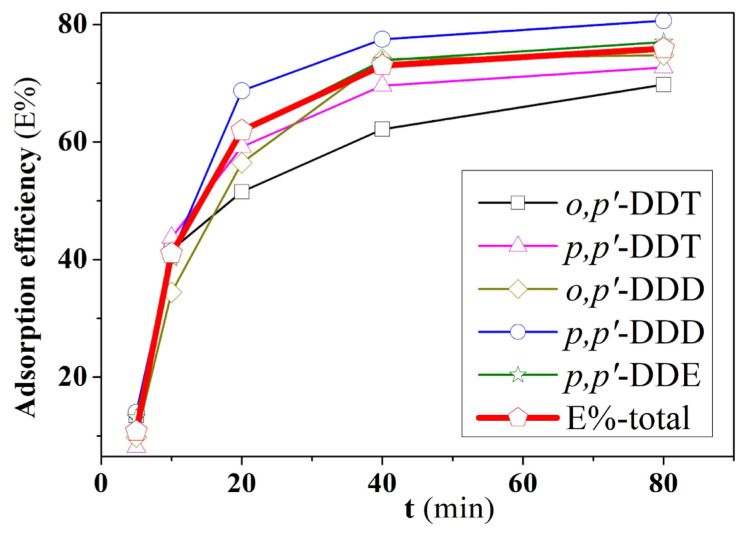
Effect of time on adsorption capacity.

The study on the effect of adsorption time can determine the equilibrium time for the adsorption process (Figure 6). All compounds showed increased adsorption efficiency when the adsorption time increased from 5 to 40 min. The period from 40 to 80 min showed a gradual decline in adsorption capacity, with no significant increase, indicating that the adsorption process gradually reached equilibrium at around 80 min (E%_total_ = 73.04 ÷ 75.92%). This suggests that the contact time between DDT, DDD, and DDE compounds and the adsorbent material affects the adsorption capacity of the material. Initially, when there are many available pores in the material, DDT, DDD, and DDE molecules can easily interact and be retained on the material’s surface. As the adsorption time increased, the adsorption capacity decreased due to the reduction of available pores, and the process reached equilibrium when most of the material’s surface was covered by DDT, DDD, and DDE molecules. Therefore, extending the adsorption time does not further enhance the material’s adsorption capacity.

#### 3.2.3. Effect of adsorbent material mass

**Figure 7:**
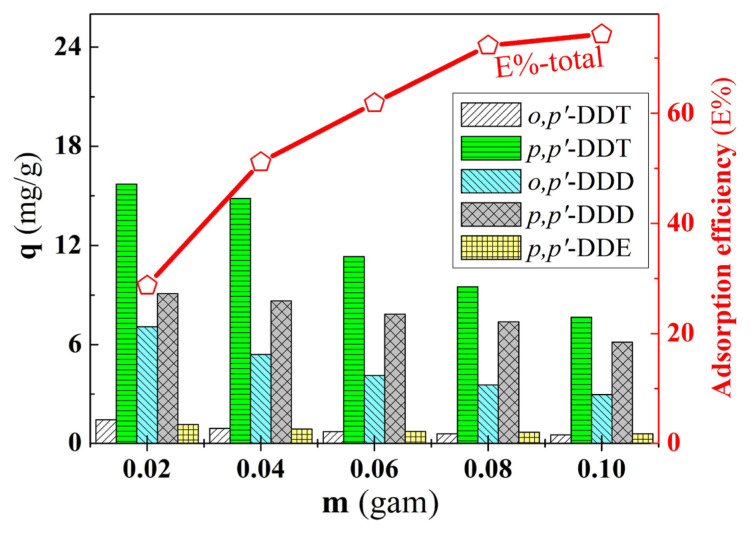
Effect of PA/BG material weight on adsorption capacity.

The study on the influence of varying the mass of PA/BG material from 0.02 to 0.10 grams on the adsorption capacity of DDT, DDD, and DDE compounds (Figure 7) over 120 min showed that the adsorption capacity for these compounds decreased, but the adsorptive efficiency increased with increasing mass. This is explained by the fact that increasing the mass of the PA/BG material increased the number of adsorption sites, thereby enhancing the retention of the adsorbate molecules by the adsorbent material. This result was consistent with the calculation formulas (2–3). The adsorption efficiency for removing DDT, DDD, and DDE compounds remained stable and did not change significantly when the material mass increased from 0.08 g (E% _total_ = 72.30%) to 0.10 grams (E% _total_ = 74.35%). This indicates that, under the conducted adsorption conditions, a mass of 0.10 g of the adsorbent material is considered optimal for the adsorption process.

#### 3.2.4. Effect of concentration of DDT, DDD, and DDE compounds

**Figure 8:**
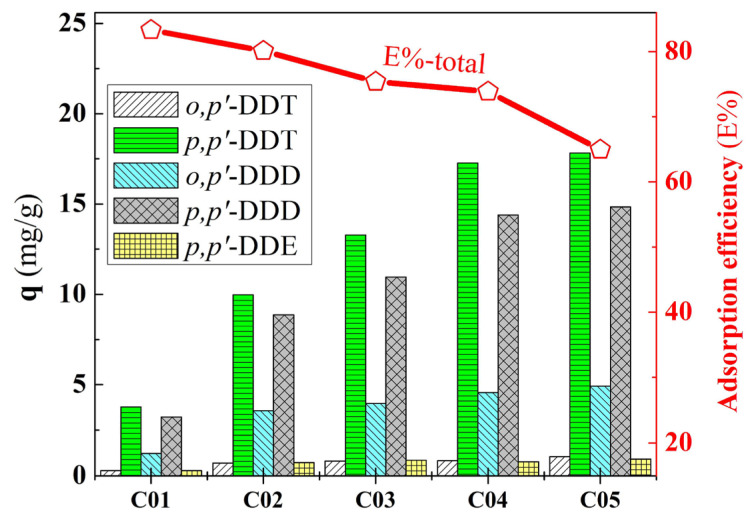
Effect of concentration on adsorption capacity.

The results in Figure 8 depict the adsorption capacity and adsorption efficiency with varying concentrations of DDT, DDD, and DDE compounds using the PA/BG composite material. Specifically, the highest adsorption efficiency (82.17%) and the lowest adsorptive capacity (8.67 mg/g) corresponded to the lowest concentrations of the adsorbates. Conversely, the lowest adsorption efficiency (64.81%) and the highest adsorptive capacity (39.48 mg/g) corresponded to the highest concentrations of the adsorbates. This indicates that at lower concentrations, the DDT, DDD, and DDE molecules have more space and better conditions to interact with the adsorbent material at its available adsorption sites. However, increasing the concentrations of the adsorbates (while keeping the mass of the adsorbent constant) reduced the ratio of available adsorption sites to the number of adsorbate molecules, leading to a decrease in the adsorption efficiency of the material.

### 3.3. Isothermal adsorption model

The Langmuir and Freundlich isotherm models were used to describe the adsorption process of *o,p′*-DDT, *p,p′*-DDT, *o,p′*-DDD, *p,p′*-DDD, and *p,p′*-DDE compounds using the PANi/bagasse composite material at a constant room temperature of 25 °C.

**Figure 9:**
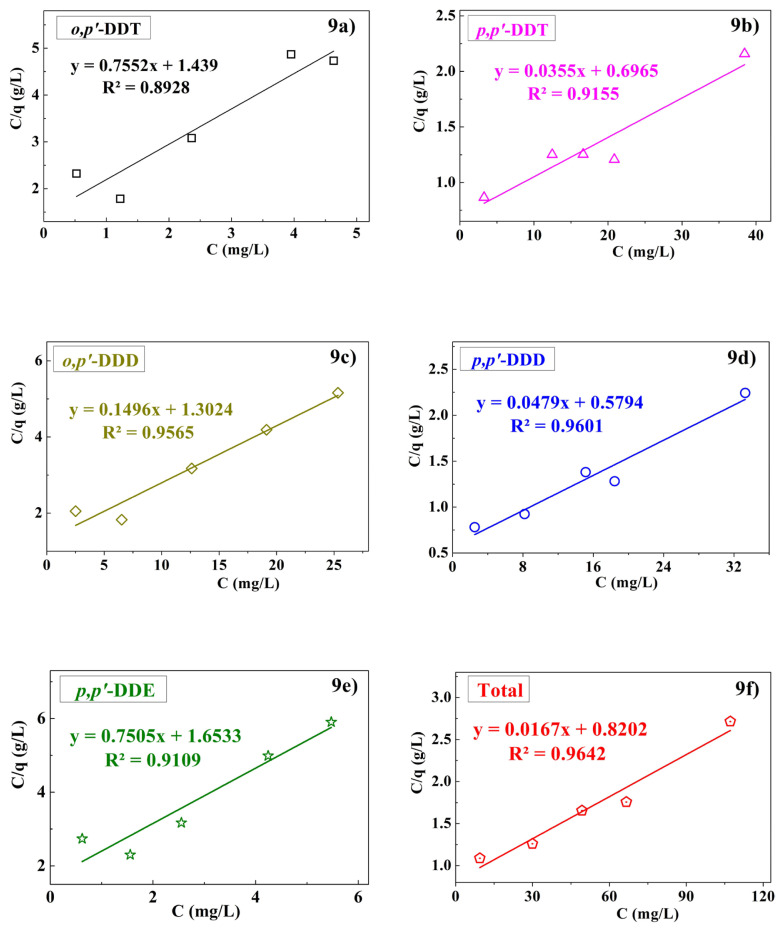
Langmuir isotherm adsorption.

The adsorption results of the five compounds and the total adsorbed substances shown in Figure 9 and Table 4 indicate that the Langmuir isotherm model is suitable for the adsorption process. This is demonstrated by the Langmuir model parameters: q_max_ = 1.0624 ÷ 28.1899 mg/g and K_L_ = 0.0203 ÷ 0.6541 L/mg, with K_L_ values within the favorable range of 0 to 1 and a relatively high R^2^ value. Notably, the separation factor R_L_, (or equilibrium parameter), which characterizes the Langmuir isotherm, was calculated using equation (**6**) and depended on the initial concentration C_0_ and K_L_ [37].


(6) $${\text{R}}_{\text{L}}=\frac{1}{1+{\text{K}}_{\text{L}}\times {\text{C}}_{0}}$$

Where: K_L_ is a constant characteristic for the Langmuir adsorption model (L/mg); **C_0_** is the initial concentration of DDT, DDD, and DDE compounds.

The value of R_L_ indicates that the adsorption process using the Langmuir model is unfavourable if R_L_ > 1, linear if R_L_ = 1, favorable if 0 < R_L_ < 1, or irreversible if R_L_ = 0.

**Figure 10:**
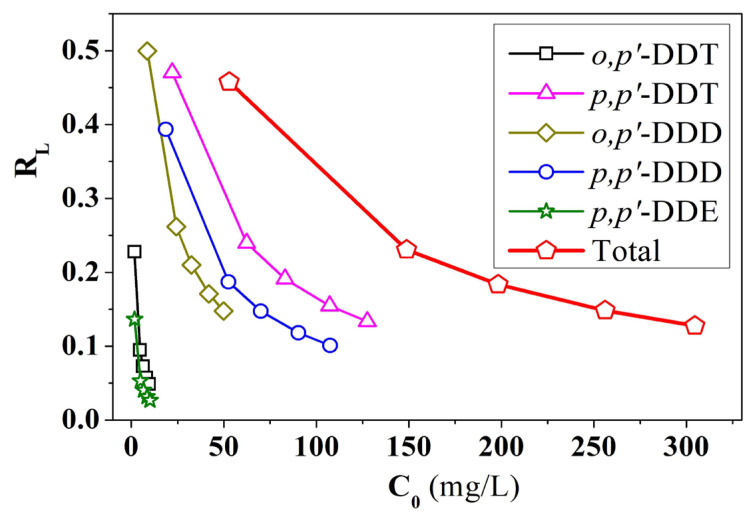
Relationship between R_L_ and initial concentration C_0_

The study of the separation factor R_L_ (Eq. 6) and the effect of R_L_ with C_0_ (Figure 10) shows that the R_L_ values range from 0 to 1, indicating favorable adsorption according to the Langmuir model. The R_L_ value decreases as the concentration of the adsorbate increases, indicating that the adsorption process becomes more favorable with higher adsorbate concentrations. This shows that the adsorption of DDT, DDD, and DDE compounds on the PA/BG material surface occurs as a homogeneous monolayer, with no interaction between adjacent adsorbed molecules until desorption. Therefore, the PA/BG adsorbent surface can only adsorb a certain number of adsorbate molecules at active sites, with the maximum adsorption capacity calculated from this model being 59.9738 mg/g.

**Figure 11:**
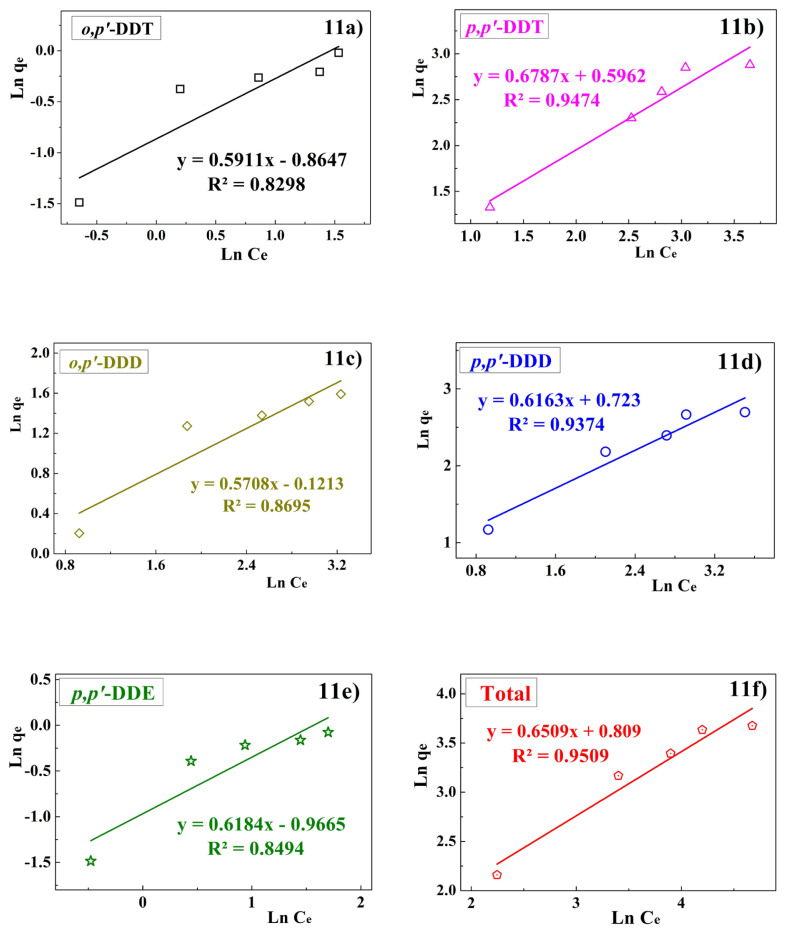
Freundlich isotherm adsorption.

The Freundlich adsorption model describes multilayer adsorption on a heterogeneous surface, making it challenging to determine the maximum adsorption capacity (q_max_). Results from applying the Freundlich isotherm (Figure 11) yielded characteristic constants of K_F_ = 0.3804 to 2.0607 and n = 1.4734 to 1.752 (with n falling within the favorable range of 0 to 10). The correlation coefficient R^2^ ranges from 0.8298 to 0.9474, suggesting the Freundlich model adequately describes the adsorption process. However, its R^2^ values are lower compared to the Langmuir model, indicating that the adsorption process using PA/BG material is better represented by the Langmuir model.

Based on previous research results on the adsorption capabilities of various materials in removing DDT, DDD, DDE, and PCBs, a comparison with the results of the material in this study (Table 5), shows that the PA/BG material demonstrates higher adsorption efficiency for DDT, DDD, and DDE compounds than previously studied materials. However, for commercial or high-cost materials such as PAC F400 [16], the effectiveness is greater than that of PA/BG. This can be seen as a positive indicator for the use of readily available and inexpensive biomass materials when combined with other potential adsorbent materials for application in current environmental pollution treatment.

## 4. Conclusion

The PANi/bagasse composite adsorbent materials were synthesized using different mass ratios of aniline/bagasse through a chemical method. The results demonstrated that these materials could adsorb and remove *o,p′*-DDT, *p,p′*-DDT, *o,p′*-DDD, *p,p′*-DDD, and *p,p′*-DDE compounds. Among the synthesized materials, those combining PANi and bagasse showed better adsorption performance than the individual components. The equilibrium adsorption time was determined to be 80 min, and the optimal adsorbent weight was 0.1 g. Two adsorption isotherm models, Langmuir and Freundlich, which were used to evaluate the adsorption mechanism of DDT, DDD, and DDE compounds using PA/BG material, indicated that the Langmuir adsorption model fits the obtained data better. The research results indicate that PANi/bagasse composite materials can be applied for the adsorption of persistent organic pollutants, utilizing waste bagasse combined with PANi. The ability of PA/BG composite materials to remove DDT, DDD, and DDE compounds from solutions provides a basis for further studies on using PA/BG materials for the removal of other chlorine-based pesticides, PCBs, and dioxins in future research.

## Figures and Tables

**Table 1:** Percentage of compounds in the initial contaminated solution.

Compounds	%
*o,p′*-DDT	3.13
*p,p′*-DDT	41.89
*o,p′*-DDD	16.39
*p,p′*-DDD	35.28
*p,p′*-DDE	3.31

**Table 2:** The synthesis yield of PANi materials with bagasse at different ratios.

Ratios of ANi/bagasse	m_ANi_ m_1_ (g)	m_BG_ m_2_ (g)	m_PANi/BG_ m_3_ (g)	Yield (%)
1/0	10.0	0	8.263	82.63
2/1	10.0	5.0	13.322	83.22
1/1	5.0	5.0	9.346	86.92
1/2	5.0	10.0	14.256	85.12

**Table 3:** Functional groups of materials in the IR spectrum.

Wavenumber *ν* (cm^−1^)			Groups a,b
BG	PANi	PANi/BG	
1054.06	NS	1113.52	C–O of glucosidic
1161.99	NS		C–O–C of pyranose
1251.69	NS	1298.58	C–O
1377.19	NS	weak	C–H aliphatic
1731.58	NS	weak	C=O
2930.44	NS	weak	C–H
3430.31	NS	3440.33	OH
NS	3444.89		>N–H
NS	1567.72	1582.04	benzoid
NS	1488.26	1486.06	quinoid
NS	1299.36	1298.58	**-**N=quinoid=N**-**
NS	1113.74	1113.52	C–N^+^
NS	814.62	795.30	C–H of benzene

a functional groups of PANi [32];

b functional groups of bagasse [33];

NS- no signal

**Table 4:** Parameters for the Langmuir and Freundlich isotherm models.

Compounds	Langmuir			Freundlich		
	R^2^	q_max_ (mg/g)	K_L_ (L/mg)	R^2^	K_F_	n
***o,p′*** **-DDT**	0.8928	1.0624	0.6541	0.8298	0.4212	1.6918
***p,p′*** **-DDT**	0.9155	28.1899	0.0509	0.9474	1.8153	1.4734
***o,p′*** **-DDD**	0.9565	6.6363	0.1157	0.8695	0.8858	1.7520
***p,p′*** **-DDD**	0.9601	20.8737	0.0827	0.9374	2.0607	1.6226
***p,p′*** **-DDE**	0.9109	1.0724	0.5640	0.8494	0.3804	1.6182
**Total**	0.9642	59.9738	0.0203	0.9509	2.2457	1.5362

**Table 5:** Comparison of the adsorption capacities of different materials for DDT, DDD, DDE, and PCBs compounds.

Materials	Adsorbed compounds	Adsorption capacity	Reference
Wood sawdust Cork wastes PAC F400	*p,p′*-DDT	69.44 mg/g 19.08 mg/g 163.90 mg/g	[15]
Silica SBA-15	DDT	6.5 μg/mg	[36]
PANi/SD/TiO_2_	PCB 28 PCB 52 PCB 101	8.09 mg/g 4.85 mg/g 3.76 mg/g	[16]
Activated carbon (AC)	DDT	10.04÷1.98 ppb	[37]
Carbon nanotube (MWNT)	DDT PCB (2-chlorobiphenyl)	47.0 mg/g 39.5 mg/g	[38]
Nano-clay	DDT PCB (2-chlorobiphenyl)	35.8 mg/g 32.9 mg/g	
Nano-alumina	DDT PCB (2-chlorobiphenyl)	31.1 mg/g 27.3 mg/g	
Polystyrene (PS) Polyethylene (PE) Polyethylene terephthalate (PET)	PCBs	0.363 ng/μg 0.574 ng/μg 0.657 ng/μg	[39]
GAC GAC-Fe_3_O_4_	dioxin	9.3 mg/g 8.3 mg/g	[40]
PANi/sawdust	DDT DDD DDE **Total**	23.50 mg/g 9.51 mg/g 13.65 mg/g **46.66 mg/g**	[25]
PANi/coir	DDT DDD DDE **Total**	23.91 mg/g 9.58 mg/g 14.15 mg/g **47.64 mg/g**	[26]
PANi/bagasse	*o,p′*-DDT *p,p′*-DDT *o,p′*-DDD *o,p′*-DDD *p,p′*-DDE **Total**	0.89 mg/g 18.83 mg/g 8.46 mg/g 16.75 mg/g 1.35 mg/g **47.28 mg/g**	This work

## Footnotes

1 Enviliance (2022). Environmental assessment report for 2016–2020 published in Vietnam [online]. Website https://enviliance.com/regions/southeast-asia/vn/report_5220 [accessed 23 May 2023].

2 Active-Oxygens (2023). Soil and groundwater remediation Daramend^®^ Reagent. [online]. Website https://active-oxygens.evonik.com/en/products-and-services/soil-and-groundwater-remediation/daramend-reagent [accessed 6 December 2023]
